# Supplementary material for: Characterization of Influenza D Virus in Danish Calves
Source: Viruses. 2022 Feb 18;14(2):423. doi: 10.3390/v14020423 (PMC8880214; doi:10.3390/v14020423)
Supplement: Supplementary file 1 [file viruses-14-00423-s001.zip › viruses-1593032-supplementary.pdf]

viruses

MDPI

Table S1. Information about collection date, number of tested animals and samples, high-throughput real-time PCR results, and IDV single-plex real-time RT-PCR results for the influenza D virus positive herds. IDV, influenza D virus; BCoV, bovine coronavirus.

		High-throughput real-time PCR analysis					Single-plex real-time RT-PCR analysis
**Herd ID**	**Collection date**	**No. of animal tested**	**Tested as pool (no. of samples in the pool)**	**Pool tested positive for**	**No. of samples tested as individual sample**	**Individual samples tested positive for**	**No. of individual samples from pool tested positive for IDV**
1	06-02-19	8	Yes (8/8)	IDV<br>*Mycoplasma*<br>*P. multocida*	4	1. IDV, *P. multocida*<br>2. IDV<br>3. IDV, *Mycoplasma*, *P. multocida*<br>4. IDV	4/4<br><br>One sample (3) sequenced<br>D/bovine/Denmark/6171103755-1/2019
1	06-03-19	4	Yes (4/4)	IDV<br>*P. multocida*<br>*T. pyogenes*	0	NA	1/4<br><br>No sample sequenced
2	01-03-19	10	Yes (10/10)	IDV<br>*M. bovis*<br>*M. haemolytica*<br>*P. multocida*	0	NA	10/10<br><br>One sample sequenced<br>D/bovine/Denmark/5246407608-2/2019
3	12-03-19	8	Yes (8/8)	IDV<br>*M. haemolytica*<br>*H. somni*<br>*P. multocida*	0	NA	3/8<br><br>One sample sequenced<br>D/bovine/Denmark/5358904609-3/2019
4	05-04-19	10	Yes (10/10)	BCoV<br>IDV<br>*M. bovis*<br>*Mycoplasma*<br>*M. haemolytica*<br>*P. multocida*<br>*T. pyogenes*	0	NA	6/10<br><br>One sample sequenced<br>D/bovine/Denmark/1173303525-4/2019
5	15-01-19	10	Yes (10/10)	IDV<br>*M. bovis*<br>*Mycoplasma*<br>*M. haemolytica*<br>*P. multocida*	0	NA	3/10<br><br>Three samples sequenced<br>D/bovine/Denmark/4559602762-5/2019<br>D/bovine/Denmark/5022903340-5/2019<br>D/bovine/Denmark/5161205135-5/2019
5	15-01-19	15	Yes (9/15)	IDV<br>*M. bovis*<br>*Mycoplasma*<br>*M. haemolytica*<br>*P. multocida*	15	1. IDV, M. *haemolytica*, *H. somni*, *P. multocida*, *T. pyogenes*<br>2. *M. bovis*, *Mycoplasma*, *M. haemolytica*, *H. somni*, *P. multocida*<br>3. IDV, *M. haemolytica*, *H. somni*, *P. multocida*<br>4. *H. somni*, *P. multocida*<br>5. IDV, *M. bovis*, *P. multocida*<br>6. IDV, *Mycoplasma*, *M. haemolytica*, *H. somni*, *P. multocida*<br>7. IDV<br>8. IDV, *P. multocida*<br>9. IDV, *Mycoplasma*, *H. somni*, *P. multocida*	7/8<br><br>One sample (10) sequenced<br>D/bovine/Denmark/5195604714-5/2019

						10. IDV, *M. haemolytica, H. somni*<br>11. Neg<br>12. IDV, *M. haemolytica, P. multocida*<br>13. IDV, *M. bovis, Mycoplasma, H. somni, P. multocida*<br>14. IDV, *M. bovis, M. haemolytica, P. multocida, T. pyogenes*<br>15. *H. somni, P. multocida*	
5	15-01-19	3	Yes (2/3)	IDV<br>*Mycoplasma*<br>*M. haemolytica*<br>*H. somni*<br>*P. multocida*	3	1. IDV<br>2. *Mycoplasma, M. haemolytica, H. somni, P. multocida*<br>3. IDV, *M. haemolytica, P. multocida*	2/3<br><br>No sample sequenced
5	20-02-19	9	Yes (9/9)	IDV<br>*M. bovis*<br>*Mycoplasma*<br>*P. multocida*	0	NA	2/9<br><br>One sample sequenced<br>D/bovine/Denmark/5195604790-5/2019
5	24-04-19	6	Yes (6/6)	IDV<br>*M. bovis*<br>*Mycoplasma*<br>*M. haemolytica*<br>*H. somni*<br>*P. multocida*	0	NA	2/6<br><br>One sample sequenced<br>D/bovine/Denmark/5224403563-5/2019
6	08-01-19	5	Yes (5/5)	IDV<br>*M. haemolytica*<br>*H. somni*<br>*P. multocida*	5	1. IDV, *T. pyogenes*<br>2. IDV, *M. bovis, Mycoplasma, M. haemolytica, H. somni, P. multocida*<br>3. *M. bovis, H. somni*<br>4. *Mycoplasma, M. haemolytica*<br>5. *P. multocida*	1/1<br><br>One sample (1) sequenced<br>D/bovine/Denmark/3313408142-6/2020
6	08-01-19	2	No	NA	2	1. IDV, *Mycoplasma, H. somni, P. multocida, T. pyogenes*<br>2. IDV, *M. haemolytica, H. somni, P. multocida*	0/0<br><br>No sample sequenced
6	05-02-19	5	Yes (5/5)	IDV<br>*M. haemolytica*<br>*P. multocida*	0	NA	0/0<br><br>No sample sequenced
6	05-02-19	5	Yes (5/5)	IDV<br>*Mycoplasma*<br>*M. haemolytica*<br>*P. multocida*	0	NA	0/0<br><br>No sample sequenced
7	08-01-20	1	No	NA	1	1. IDV	1/1<br><br>No sample sequenced
7	05-02-20	5	Yes (5/5)	IDV<br>*M. bovis*<br>*Mycoplasma*<br>*M. haemolytica*<br>*P. multocida*	0	NA	0/0<br><br>No sample sequenced
7	05-02-20	5	Yes (5/5)	IDV<br>*M. bovis*<br>*Mycoplasma*	0	NA	0/0<br><br>No sample sequenced

				M. haemolytica<br>*P. multocida*			
7	05-02-20	5	Yes (5/5)	IDV<br>*M. haemolytica*<br>*P. multocida*	0	NA	0/0<br>No sample sequenced
7	12-03-20	5	Yes (5/5)	BCoV<br>IDV<br>*Mycoplasma*<br>*M. haemolytica*<br>*P. multocida*	0	NA	5/5<br>One sample sequenced<br>D/bovine/Denmark/3727003200-7/2020
8	06-03-20	5	Yes (5/5)	IDV<br>*M. bovis*<br>*Mycoplasma*<br>*M. haemolytica*<br>*H. somni*<br>*P. multocida*<br>*T. pyogenes*	1	1. Neg	3/5<br>One sample sequenced<br>D/bovine/Denmark/5256205576-8/2020
9	17-12-19	1	No	NA	1	1. IDV, *H. somni, P. multocida*	1/1<br>No sample sequenced
9	17-12-19	5	Yes (5/5)	IDV<br>*M. bovis*<br>*Mycoplasma*<br>*M. haemolytica*<br>*P. multocida*<br>*T. pyogenes*	2	1. *M. haemolytica, P. multocida*<br>2. *M. bovis, Mycoplasma, H. somni*	0/0<br>No sample sequenced
9	17-12-19	5	Yes (5/5)	IDV<br>*P. multocida*	2	1. *M. bovis, Mycoplasma, H. somni, P. multocida*<br>2. *M. haemolytica*	0/0<br>No sample sequenced
9	11-02-20	5	Yes (5/5)	IDV<br>*M. bovis*<br>*Mycoplasma*<br>*P. multocida*	0	NA	4/5<br>One sample sequenced<br>D/bovine/Denmark/5995505714-9/2020
9	11-02-20	5	Yes (5/5)	IDV<br>*M. bovis*<br>*Mycoplasma*<br>*P. multocida*	0	NA	0/0<br>No sample sequenced
9	05-03-20	5	Yes (5/5)	IDV<br>*M. bovis*<br>*Mycoplasma*<br>*M. haemolytica*<br>*H. somni*<br>*P. multocida*<br>*T. pyogenes*	0	NA	0/0<br>No sample sequenced
10	16-01-20	1	No	NA	1	1. IDV, *Mycoplasma, H. somni, P. multocida*	1/1<br>No sample sequenced
10	16-01-20	6	Yes (4/6)	IDV<br>*M. bovis*<br>*Mycoplasma*	6	1. *Mycoplasma, H. somni, P. multocida*<br>2. IDV, *M. haemolytica, H. somni, P. multocida*<br>3. IDV, *M. bovis, Mycoplasma, M. haemolytica, H. somni*	1/1<br>One sample sequenced

				M. haemolytica<br>*H. somni*<br>*P. multocida*		4. *M. bovis, M. haemolytica, H. somni, P. multocida*<br>5. Neg<br>6. *M. bovis, H. somni, P. multocida*	D/bovine/Denmark/1052304114-10/2020
10	10-03-20	4	Yes (4/4)	IDV<br>*M. bovis*<br>*Mycoplasma*<br>*M. haemolytica*<br>*H. somni*<br>*P. multocida*	0	NA	0/0<br><br>No sample sequenced
10	10-03-20	5	Yes (5/5)	IDV<br>*Mycoplasma*<br>*M. haemolytica*<br>*H. somni*<br>*P. multocida*	0	NA	0/0<br><br>No sample sequenced
11	09-10-19	5	Yes (5/5)	IDV<br>*Mycoplasma*<br>*H. somni*	0	NA	4/5<br><br>One sample sequenced<br>D/bovine/Denmark/3058506689-11/2019
12	16-01-19	10	Yes (10/10)	IDV<br>*M. bovis*<br>*Mycoplasma*<br>*M. haemolytica*<br>*P. multocida*<br>*T. pyogenes*	0	NA	6/10<br><br>No sample sequenced
13	03-11-15	0	No	NA	0	NA	1/1<br>One sample sequenced<br>D/bovine/Denmark/47398-3622/2015

Table S2. Accession numbers for the sequences of this study.

Strain	PB2	PB1	P3	HEF	NP	P42	NS
D/bovine/Denmark/6171103755-1/2019	OM468189	OM468201	OM468224	OM468243	OM468260	OM468270	OM468297
D/bovine/Denmark/5246407608-2/2019	OM468185	OM468202	OM468217	OM468246	OM468253	OM468265	OM468283
D/bovine/Denmark/5358904609-3/2019	OM468188	OM468203	OM468227	OM468247	OM468259	OM468269	OM468281
D/bovine/Denmark/1173303525-4/2019	OM468187	OM468207	OM468226	OM468242	OM468254	OM468267	OM468282
D/bovine/Denmark/4559602762-5/2019	OM468196	OM468211	OM468218	OM468240	OM468258	OM468274	OM468290
D/bovine/Denmark/5022903340-5/2019	OM468193	OM468208	OM468219	OM468237	OM468256	OM468275	OM468287

D/bovine/Denmark/5161205135-5/2019	OM468194	OM468209	OM468220	OM468239	OM468257	OM468273	OM468288
D/bovine/Denmark/5195604714-5/2019	OM468195	OM468210	OM468221	OM468238	OM468255	OM468276	OM468289
D/bovine/Denmark/5195604790-5/2019	OM468190	OM468212	OM468225	OM468245	OM468261	OM468272	OM468284
D/bovine/Denmark/5224403563-5/2019	OM468191	OM468213	OM468223	OM468244	OM468262	OM468271	OM468285
D/bovine/Denmark/3313408142-6/2020	OM468186	OM468206	OM468228	OM468241	OM468264	OM468266	OM468286
D/bovine/Denmark/3727003200-7/2020	OM468197	OM468214	OM468229	OM468233	OM468252	OM468277	OM468292
D/bovine/Denmark/5256205576-8/2020	OM468198	OM468215	OM468230	OM468234	OM468249	OM468278	OM468293
D/bovine/Denmark/5995505714-9/2020	OM468200	OM468216	OM468232	OM468236	OM468251	OM468280	OM468295
D/bovine/Denmark/1052304114-10/2020	OM468199	OM468205	OM468231	OM468235	OM468250	OM468279	OM468294
D/bovine/Denmark/3058506689-11/2019	OM468192	OM468204	OM468222	OM468248	OM468263	OM468268	OM468291
D/bovine/Denmark/47398-3622/2015	-	-	-	OM468296	-	-	-

Table S3. Predicted N-linked glycosylation sites of HEF for D/660 influenza D viruses (from Figure 1).

**D/660 Sequences**	**Amino acid positions**										
	**28**	**54**	**133**	**146**	**249**	**254**	**346**	**390**	**513**	**562**	**613**
	**NESF**	**NVTK**	**NWSP**	**NWTQ**	**NKTA**	**NTTG**	**NATE**	**NDTV**	**NDTN**	**NISM**	**NGSA**
D/bovine/Italy/35490/2019	+	+++	---	+	++	/	+	-	+	-	+
D/bovine/Italy/28300/2019	+	+++	---	+	NRTA ++	/	+	-	+	-	+
D/bovine/Italy/28145/2019	+	+++	---	+	++	/	+	-	+	-	+
D/bovine/Italy/45097/2019	+	+++	---	+	++	/	+	-	+	-	+
D/bovine/Denmark/3727003200-7/2020-03-12	+	+++	---	+	++	/	+	-	+	-	+
D/bovine/Italy/19RS176-11/2018	+	+++	---	+	++	/	+	-	+	-	+
D/bovine/Denmark/5995505714-9/2020-02-11	+	+++	---	+	++	/	+	-	+	-	+

D/bovine/Denmark/1052304114-10/2020-01-16	+	+++	---	+	++	/	+	-	+	-	+
D/bovine/Denmark/5256205576-8/2020-03-06	+	+++	---	+	++	/	+	-	+	-	+
D/bovine/Quebec/5E-H/2018	+	+++	---	+	++	-	+	-	+	-	+
D/bovine/Quebec/3E-H/2018	+	+++	---	+	++	/	+	-	+	-	+
D/bovine/Quebec/1M-H/2019	+	+++	---	+	/	/	+	-	+	-	+
D/bovine/Mississippi/C00009P/2014	+	+++	---	+	++	/	+	-	+	-	+
D/bovine/Mississippi/C00002N/2014	+	+++	---	+	++	/	+	-	+	-	+
D/bovine/Nebraska/9-5/2012	+	+++	---	+	++	/	+	-	+	-	+
D/bovine/Oklahoma/660/2013	+	+++	---	+	NKTX ++	/	+	-	+	-	+
D/bovine/Mexico/S7/2015	+	+++	---	+	++	/	+	-	+	-	+
D/bovine/Mexico/S56/2015	+	+++	---	+	++	/	+	-	+	-	+
D/bovine/Quebec/3M-B/2020	+	+++	---	+	++	/	+	-	+	-	+
D/bovine/France/2986/2012	+	+++	---	+	/	/	+	-	/	-	+

+ indicates prediction of an N-glycosylated site. - indicates a non-glycosylated site. / indicates no prediction of a glycosylated site.
Definition according to https://services.healthtech.dtu.dk/service.php?NetNGlyc-1.0:
+ Potential > 0.5, ++ Potential > 0.5 and Jury agreement (9/9) or Potential > 0.75, +++ Potential > 0.75 and Jury agreement, ++++ Potential > 0.90 and Jury agreement.
- Potential < 0.5, - - Potential < 0.5 and Jury agreement (all nine < 0.5), - - - Potential < 0.32 and Jury agreement.

Table S4. Predicted N-linked glycosylation sites of HEF for D/OK influenza D viruses (from Figure 1).

**D/OK Sequences**	**Amino acid positions**									
	**28**	**54**	**133**	**146**	**249**	**346**	**390**	**513**	**562**	**613**
	**NESF**	**NVTK**	**NWSP**	**NWTQ**	**NKTA**	**NATE**	**NDTV**	**NDTN**	**NLSM**	**NGSA**
D/bovine/Italy/16RS2359/2016 (+1)	+	+++	---	+	++	+	-	+	-	+
D/bovine/Italy/4128547/2018 (+1)	+	+++	---	+	++	+	-	+	-	+
D/bovine/Italy/18RS182-14/2017 (+1)	+	+++	---	+	++	+	-	+	-	+
D/bovine/Italy/59542/2018 (+1)	+	+++	---	+	++	+	-	+	-	+
D/bovine/Italy/50543-3/2018 (+1)	+	+++	---	+	++	+	-	+	-	+
D/swine/Italy/218655/2017 (+1)	+	+++	---	+	++	+	-	/	-	+
D/bovine/Italy/37760/2019 (+1)	+	/	---	NWTH +	++	+	-	+	-	+
D/swine/Italy/173287-4/2016 (+1)	+	+++	---	++	+++	+	-	+	-	+
D/swine/Italy/254578/2015 (+1)	+	+++	---	+	++	+	-	-	-	+

D/bovine/Italy/31525/2018 (+1)	+	+++	---	+	++	+	-	+	-	+
D/bovine/Italy/18RS182-13/2017 (+1)	+	+++	---	+	++	+	-	+	-	+
D/bovine/Italy/18RS182-8/2017 (+1)	+	+++	---	+	++	+	-	+	-	+
D/bovine/Italy/16RS2581-9/2016 (+1)	+	+++	---	+	++	+	-	+	-	+
D/bovine/France/5920/2014 (+1)	+	+++	---	+	++	+	-	+	-	+
D/bovine/Italy/46484/2015 (+1)	+	+++	---	+	++	+	-	+	-	+
D/bovine/Denmark/5195604790-5/2019-02-20	+	/	---	+	++	+	-	+	-	+
D/bovine/Denmark/5224403563-5/2019-04-24	+	+++	---	+	++	+	-	+	-	+
D/bovine/Denmark/6171103755-1/2019-02-06	+	+++	---	+	++	+	-	+	-	+
D/bovine/Denmark/1173303525-4/2019-04-05	+	+++	---	+	++	+	-	+	-	+
D/bovine/Denmark/3313408142-6/2020-01-08	+	+++	---	+	++	+	-	+	-	+
D/bovine/Denmark/3058506689-11/2019-10-09	+	+++	---	+	++	+	-	+	-	+
D/bovine/Denmark/4559602762-5/2019-01-15	+	+++	---	+	++	+	-	+	-	+
D/bovine/Denmark/5195604714-5/2019-01-15	+	+++	---	+	++	+	-	+	-	+
D/bovine/Denmark/5161205135-5/2019-01-15	+	+++	---	+	++	+	-	+	-	+
D/bovine/Denmark/5022903340-5/2019-01-15	+	+++	---	+	++	+	-	+	-	+
D/bovine/Denmark/5358904609-3/2019-03-12	+	+++	---	+	++	+	-	+	-	+
D/bovine/Denmark/5246407608-2/2019-03-01	+	+++	---	+	++	+	-	/	-	+
D/bovine/Denmark/47398-3622/2015-11-03	+	+++	---	+	++	+	-	/	-	+
D/bovine/Italy/1/2014 (+1)	+	+++	---	+	++	+	-	+	-	+
D/swine/Italy/18VIR6833/2015 (+1)	+	+++	---	+	++	+	-	+	-	+
D/bovine/Italy/18RS182-16/2018 (+1)	+	+++	---	+	++	+	-	+	-	+
D/bovine/Italy/18RS182-10/2017 (+1)	+	+++	---	+	++	+	-	+	-	+
D/swine/Italy/354017/2015 (+1)	+	+++	---	+	++	/	-	+	-	+
D/bovine/Northern Ireland/24280/2017 (+1)	+	+++	---	+	++	+	-	+	-	+
D/bovine/Italy/40574/2018 (+1)	+	+++	---	+	++	+	-	+	-	+
D/bovine/Mississippi/C00030P/2014 (+1)	+	+++	---	+	NKTT ++	+	-	+	-	+
D/bovine/Mississippi/C00020N/2014 (+1)	+	+++	---	+	NKTT ++	+	-	+	-	+
D/bovine/Mississippi/C00135N/2014 (+1)	+	+++	---	+	NKTT ++	+	-	+	-	+
D/bovine/Mississippi/C00162N/2015 (+1)	+	+++	---	+	NKTT ++	+	-	+	-	+
D/bovine/Mississippi/C00165P/2015 (+1)	+	+++	---	+	NKTT ++	+	-	+	NISM -	+
D/bovine/Kansas/1-35/2010 (+1)	+	+++	---	+	NKTT ++	+	-	+	NISM -	+
D/bovine/Minnesota/729/2013 (+1)	+	+++	---	+	NKTT ++	+	-	+	NISM -	+
D/bovine/Minnesota/628/2013 (+1)	+	+++	---	+	NKTT ++	+	-	+	NISM -	+
D/bovine/Guangdong/YC/2017 (+1)	+	+++	---	+	NKTT ++	/	-	+	NISM -	+
D/bovine/Guangdong/SK/2018 (+1)	+	+++	---	+	NKTT ++	+	-	+	NISM -	+

D/bovine/Shandong/Y217/2014 (+1)	+	+++	---	+	NKTT<br>++	+	-	+	NISM<br>-	+
D/bovine/Shandong/Y125/2014 (+1)	+	+++	---	+	NKTT<br>++	+	-	+	NISM<br>-	+
D/bovine/Mississippi/C00148N/2014 (+1)	+	/	---	+	++	+	-	+	NISM<br>-	+
D/bovine/Texas/72/2017 (+1)	+	+++	---	+	++	+	-	+	NISM<br>-	+

+ indicates prediction of an N-glycosylated site. - indicates a non-glycosylated site. / indicates no prediction of a glycosylated site.
Definition according to https://services.healthtech.dtu.dk/service.php?NetNGlyc-1.0:
+ Potential > 0.5, ++ Potential > 0.5 and Jury agreement (9/9) or Potential > 0.75, +++ Potential > 0.75 and Jury agreement, ++++ Potential > 0.90 and Jury agreement.
- Potential < 0.5, - - Potential < 0.5 and Jury agreement (all nine < 0.5), - - - Potential < 0.32 and Jury agreement.
